# The use of apps in public mental health: assessing needs and re-developing programs for integration into society

**DOI:** 10.1192/j.eurpsy.2025.92

**Published:** 2025-08-26

**Authors:** V. J. J. A. Buwalda

**Affiliations:** Psychiatry, GGD, Amsterdam, Netherlands

## Abstract

**Abstract:**

In the last decennium there is an explosion of medical devices to help patients in their fight against their psychiatric disorder and if possible to take more control and responsibilities over their own lives. In our research on measurement instruments it was doubted that patients were able to use computers and apps. Happell, 2009, addressed that psychiatric patients were very interested in using apps and also were positive being partners in the development of apps that could enhance their quality of life. Their attitude towards apps in clinical practice was more positive than their clinicians who were afraid of being judged on their performance and are more hesitant (Buwalda, et al., 2015). 10-15 years later millions of patients and clinicians are using applications and other medical devices to enlighten their lives.

But what about the most vulnerable citizens, our patients, in the cities in the context of urbanisation. In Amsterdam the public mental health services developed the self-sufficiency matrix (SSM) to gain insight in the peoples individual possibilities. A measure that insights the adaptation to the complex city life and how they can take care of themselves in this ever changing world. How do they feel about using apps or their professionals in daily clinical practice?

This presentation is about the history of the development of a mental health app for psychiatric patients in the city to be used by the professionals. Through a small pilot the presenter will a show the process of development of the SSM-app that gives insight in the wellbeing of the most vulnerable and their needs. We will also discuss the challenges and user-friendliness of the SSM- app in our PMH to make our work more digital proof.

**Disclosure of Interest:**

None Declared

